# Metabolic Syndrome and High-Obesity-Related Indices Are Associated with Poor Cognitive Function in a Large Taiwanese Population Study Older than 60 Years

**DOI:** 10.3390/nu14081535

**Published:** 2022-04-07

**Authors:** Szu-Han Huang, Szu-Chia Chen, Jiun-Hung Geng, Da-Wei Wu, Chien-Hsun Li

**Affiliations:** 1 Department of Post Baccalaureate Medicine, Kaohsiung Medical University, Kaohsiung 807, Taiwan; gavin40708123@gmail.com; 2 Department of Internal Medicine, Kaohsiung Municipal Siaogang Hospital, Kaohsiung Medical University, Kaohsiung 812, Taiwan; scarchenone@yahoo.com.tw (S.-C.C.); u8900030@yahoo.com.tw (D.-W.W.); 3 Division of Nephrology, Department of Internal Medicine, Kaohsiung Medical University Hospital, Kaohsiung Medical University, Kaohsiung 807, Taiwan; 4 Faculty of Medicine, College of Medicine, Kaohsiung Medical University, Kaohsiung 807, Taiwan; 5 Research Center for Environmental Medicine, Kaohsiung Medical University, Kaohsiung 807, Taiwan; 6 Department of Urology, Kaohsiung Municipal Siaogang Hospital, Kaohsiung Medical University, Kaohsiung 812, Taiwan; u9001090@gmail.com; 7 Department of Urology, Kaohsiung Medical University Hospital, Kaohsiung Medical University, Kaohsiung 807, Taiwan; 8 Division of Pulmonary and Critical Care Medicine, Department of Internal Medicine, Kaohsiung Medical University Hospital, Kaohsiung Medical University, Kaohsiung 807, Taiwan; 9 Department of Neurology, Kaohsiung Medical University Hospital, Kaohsiung Medical University, Kaohsiung 807, Taiwan; 10 Department of Neurology, Kaohsiung Municipal Siaogang Hospital, Kaohsiung Medical University, Kaohsiung 812, Taiwan; 11 Integrated Center of Healthy and Long-Term Care, Kaohsiung Municipal Siaogang Hospital, Kaohsiung Medical University, Kaohsiung 812, Taiwan

**Keywords:** metabolic syndrome, obesity-related indices, Mini-Mental State Examination, cognitive impairment

## Abstract

Metabolic syndrome (MetS) is prevalent in Taiwan; however, the association between MetS and cognitive function is unclear. The aim of this study was to explore the associations between MetS, its components, and obesity-related indices with cognitive function in a large Taiwanese cohort. We enrolled a total of 28,486 participants who completed the Mini-Mental State Examination (MMSE) questionnaire, which was used to evaluate cognitive function. MetS was defined according to the NCEP-ATP III guidelines and modified criteria for Asians. Ten obesity-related indices were also evaluated: body mass index (BMI), abdominal volume index (AVI), body adiposity index (BAI), waist–hip ratio (WHR), a body shape index (ABSI), lipid accumulation product, waist-to-height ratio (WHtR), conicity index (CI), body roundness index (BRI), and triglyceride glucose index. The prevalence of MetS and its components (except for hypertriglyceridemia) and the number of MetS components increased while the cognitive impairment worsened (from MMSE ≥ 24, 18–23 to 0–17). In addition, increases in all obesity-related index values were associated with a decline in cognitive function (from MMSE ≥ 24, 18–23 to 0–17, ANOVA *p* < 0.001). Multivariable analysis showed that MetS (*p* = 0.002), abdominal obesity (*p* < 0.001), low high-density lipoprotein cholesterol (*p* = 0.004), and hyperglycemia (*p* = 0.012) were significantly associated with a low MMSE score. Further, participants with high BMI (*p* = 0.001), WHR (*p* < 0.001), WHtR (*p* < 0.001), BRI (*p* < 0.001), CI (*p* < 0.001), BAI (*p* < 0.001), AVI (*p* < 0.001), and ABSI (*p* < 0.001) values were significantly associated with a low MMSE score. Our results show that MetS and its components (except for hypertriglyceridemia and high blood pressure) may lead to cognitive impairment, and that high values of obesity-related indices were associated with poor cognitive function.

## 1. Introduction

As the global population continues to age, the effect on cognitive function will have an increasing impact on the quality of life and socio-economic costs, which will further increase the burden on families and medical care systems [1,2]. Therefore, the issue of cognitive function has received increasing attention. Mild cognitive impairment (MCI) is a transitional clinical state defined as cognitive impairment that is more severe than normal age-related cognitive impairment but not as severe as dementia [3]. Epidemiological studies have reported prevalence rates of MCI from 2.8 to 17.5% in Europe and North America to 5.4 to 25.0% in different parts of China [4]. In addition, a meta-analysis of 34 studies conducted by the American Academy of Neurology reported estimated prevalence rates of 6.7% for those aged 60–64 years, 8.4% for those aged 65–69 years, 10.1% for those aged 70–74 years, 14.8% for those aged 75–79 years, and 25.2% for those aged 80–84 years [5]. The pathogenesis of MCI has been reported to be similar to various kinds of dementia, including β amyloid-deposition for Alzheimer’s disease, synucleinopathy for Lewy body dementia and Parkinson disease dementia, and tauopathy for several neurodegenerative diseases [6]. MCI can be diagnosed clinically, and therefore a brief and comprehensive diagnostic tool is important. The Mini-Mental State Examination (MMSE) is the most widely used brief screening tool for cognition disorders [7], with a score < 24 indicating cognitive impairment [8]. The risk factors for MCI include aging, vascular risk factors (e.g., hypertension, midlife diabetes and obesity), history of stroke or heart disease, apolipoprotein E epsilon 4 genotype, vitamin D deficiency, prior critical illness (e.g., sepsis), sleep-disordered breathing, and lower educational level [9]. Many studies have reported an association between MCI and a higher risk of progressing to dementia [5], and therefore it is important to detect the risk factors for MCI as early as possible.

Metabolic syndrome (MetS) comprises a cluster of cardiovascular risk factors, including abdominal obesity, dyslipidemia, hyperglycemia, and hypertension [10]. MetS has been associated with a higher risk of developing type 2 diabetes, lipid disorders, cardiovascular disease, hepatic steatosis, and circulatory disorders [11]. Anthropometric indices including body mass index (BMI), abdominal volume index (AVI), body adiposity index (BAI), waist–hip ratio (WHR), a body shape index (ABSI), lipid accumulation product (LAP), waist-to-height ratio (WHtR), conicity index (CI), body roundness index (BRI), and triglyceride glucose index (TyG index) have been shown to be good predictors of MetS [12]. Moreover, they can easily be calculated using fasting glucose, triglyceride (TG) level, body weight (BW), body height (BH), hip circumference (HC), and waist circumference (WC). Previous studies have also shown associations between these indices and albuminuria [13], lung function [14], osteoporosis [15], peripheral artery occlusive disease [16], and fatty liver [17]. 

Several studies have investigated the association between MetS and cognitive function [18]; however, the results have been inconsistent, with one study suggesting a weak association [19] and another reporting no association [18]. Therefore, the aim of this study was to explore the associations between MetS, its components, and obesity-related indices with cognitive function in a large Taiwanese cohort from the Taiwan Biobank (TWB).

## 2. Materials and Methods

### 2.1. Ethics Statement

This study followed the Declaration of Helsinki, and was approved by the Institutional Review Board of Kaohsiung Medical University Hospital (KMUHIRB-E(I)-20210058). Ethical approval for the TWB was granted by the Institutional Review Board on Biomedical Science Research, Academia Sinica, Taiwan, and the Ethics and Governance Council of the TWB. All participants provided written informed consent. 

### 2.2. TWB and Study Variables

The TWB was established by the Ministry of Health and Welfare in Taiwan to address the aging population and longer average lifespan, promote health care, and prevent chronic diseases. It contains medical, genetic, and lifestyle data of people 30 to 70 years of age living in the community with no history of cancer [20,21]. 

All participants enrolled in the TWB complete a questionnaire during an in-person interview, and personal information including age, sex, lifestyle factors including exercise, and medical history (including diabetes mellitus [DM] and hypertension) are recorded. For the purpose of this study, “exercise” was defined as participating in a leisure activity but not occupational activity. The leisure activities included but were not limited to playing a sport, hiking, swimming, yoga, jogging, riding a bicycle, and exercise-based computer games. Regular exercise was defined as spending a minimum of 30 min participating in one of these activities at least three times per week. 

All participants enrolled in the TWB also undergo a physical examination during which WC, HC, BH, and BW are recorded. All participants also provide blood samples for laboratory data, including fasting glucose, hemoglobin, TGs, total cholesterol, high-density lipoprotein (HDL)-cholesterol, low-density lipoprotein (LDL)-cholesterol, uric acid, and estimated glomerular filtration rate (eGFR), which was calculated using the 4-variable Modification of Diet in Renal Disease study equation [22]. 

**Figure 1:**
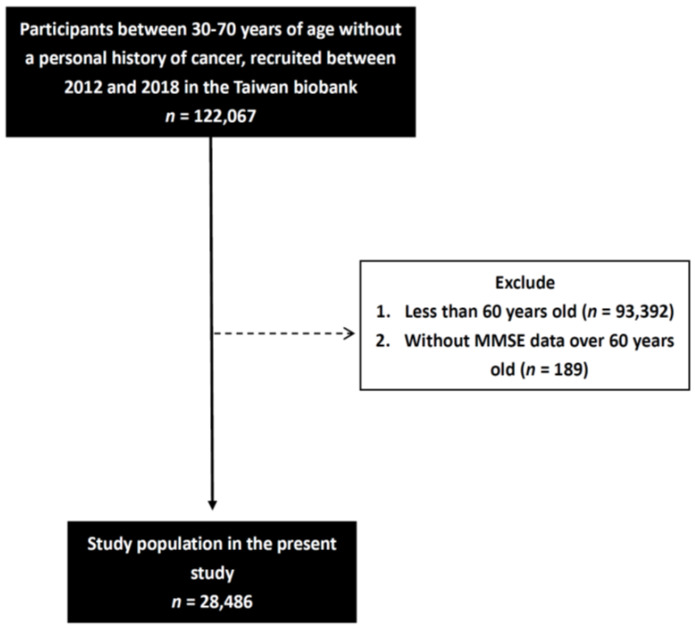
Flowchart of study population.

A total of 122,067 participants were included in the baseline study, and those older than 60 years were invited to take the MMSE (*n* = 28,675). Of these participants, 28,486 completed the MMSE questionnaire and were included in this study (Figure 1). 

### 2.3. Evaluation of Cognitive Function

We assessed the cognitive function of the participants using the MMSE [4]. The MMSE contains five subscales: orientation to time and space, 0–10 points; G2 registration, 0–3 points; attention and calculation, 0–3 points; recall 0–3 points; and language, 0–11 points. The total MMSE score was calculated as the sum of the subscores, and ranged from 0 to 30. A lower score indicated worse cognitive function. 

### 2.4. Definition of MetS 

We used the NCEP-ATP III guidelines [23] and modified criteria for Asians [24] to define MetS in this study, as the presence of three or more of the following: (1) hyperglycemia (fasting whole-blood glucose concentration ≥ 100 mg/dL) or a diagnosis of DM; (2) systolic blood pressure (BP) ≥ 130 mmHg, diastolic BP ≥ 85 mmHg, a diagnosis of hypertension, or receiving hypertensive treatment; (3) HDL-cholesterol level < 50 and < 40 mg/dL for women and men, respectively; (4) TG level ≥ 150 mg/dL; (5) abdominal obesity (WC ≥ 80 and ≥90 cm for women and men, respectively). 

### 2.5. Calculation of Obesity-Related Indices

BMI was calculated as:

BMI = BW (kg)/BH^2^ (m)

WHR was calculated as:

WHR = WC (cm)/HC (cm)

WHtR was calculated as:

WHtR = WC (cm)/BH (cm)

BRI was calculated as [25]:

$$\mathrm{BRI}=364.2-365.5\times \sqrt{1-({\frac{\mathrm{WC}\left(m\right)}{\pi \times \mathrm{BH}\left(m\right)}}^{2})}$$

CI was calculated using the Valdez equation based on BW, BH, and WC as [26]:

$$\mathrm{CI}=\frac{\mathrm{WC}\left(m\right)}{0.109\times \sqrt{\frac{\mathrm{BW}\left(\mathrm{kg}\right)}{\mathrm{BH}\left(m\right)}}}$$

BAI was calculated according to the method of Bergman and colleagues as [27,28]:

$$\mathrm{BAI}=\frac{\mathrm{HC}\left(\mathrm{cm}\right)}{\mathrm{BH}{\left(m\right)}^{\frac{3}{2}}}-18$$

$$\mathrm{AVI}=\frac{2\times \left(\mathrm{WC}{\left(\mathrm{cm}\right)}^{2}\right)+0.7\times {\left(\mathrm{WC}\left(\mathrm{cm}\right)-\mathrm{HC}\left(\mathrm{cm}\right)\right)}^{2}}{1000}$$

ABSI was calculated as [29]:

ABSI = WC (m)/[BMI^2/3^(kg/m^2^) × BH^1/2^(m)]

LAP was calculated as [30]:

LAP=(WC(cm)−65)×TG(mmol/L) in males and

LAP=(WC(cm)−58)×TG(mmol/L) in females

TyG index = Ln [fasting TG (mg/dL) × fasting plasma glucose (mg/dL)/2] [31].

### 2.6. Statistical Analysis

Data are presented as mean ± standard deviation or percentage. The chi-squared test was used to explore differences between categorical variables, and the independent *t*-test was used for continuous variables. Multiple comparisons among groups according to the severity of cognitive impairment were performed using one-way analysis of variance followed by a Bonferroni-adjusted post hoc test. Due to conducting the *t*-test and one-way analysis of variance (=general linear model) many times, the multiple-testing-adjusted corrections of *p* values were computed by stepdown Bonferroni. Significant variables in univariable analysis, MetS, and each obesity-related index were entered into multivariable analysis. Multivariable linear regression analysis was performed to explore associations between MetS and obesity-related indices with MMSE. *p* values < 0.05 were considered to be statistically significant. All statistical analyses were conducted using SPSS version 19.0 for Windows (SPSS Inc., Chicago, IL, USA).

## 3. Results

The mean age of the 28,486 enrolled participants was 63.9 ± 2.8 years old, and included 11,166 males and 17,320 females. The participants were stratified into two groups according to MMSE ≥ 24 (*n* = 26,358; 92.5%) or MMSE < 24 (*n* = 2128; 7.5%).

### 3.1. Comparison of Clinical Characteristics among the Participants According to Total MMSE Scores ≥ 24 or <24

A comparison of the clinical characteristics between the participants with MMSE ≥ 24 or <24 is shown in Table 1. Compared to the participants with MMSE ≥ 24, those with MMSE < 24 were older, predominantly female, had higher prevalence rates of DM and hypertension, lower prevalence of smoking history, lower prevalence of regular exercise habit, lower education status, lower diastolic BP, lower BH, higher WC, high HC, higher fasting glucose, lower hemoglobin, higher TG, and lower HDL-cholesterol. Regarding MetS and its components, and the obesity-related indices, the participants with MMSE < 24 had high prevalence of MetS, abdominal obesity, low HDL-cholesterol, hyperglycemia, high BP, and higher levels of all obesity-related indices, including BMI, WHR, WHtR, BRI, CI, BAI, AVI, ABSI, LAP, and TyG index.

### 3.2. Association between MetS and Its Components, and the Values of Obesity-Related Indices According to the Severity of Cognitive Impairment

Table 2 shows the prevalence of MetS and its components, and the values of obesity-related indices according to the severity of cognitive impairment in study participants. The prevalence of MetS and its components (except for hypertriglyceridemia) and MetS numbers increased with the severity of cognitive impairment (from MMSE ≥ 24, 18–23 to 0–17). In addition, the values of all obesity-related indices increased with the severity of cognitive impairment (from MMSE ≥ 24, 18–23 to 0–17, ANOVA *p* < 0.001). There was a significant trend for stepwise increases in all obesity-related indices.

### 3.3. Association of MetS and Its Components with MMSE

The factors associated with MMSE in the study participants in univariable linear regression analysis are shown in Table 3. The results show that old age, female sex, DM, hypertension, no smoking history, no regular exercise habits, low education level, not living alone, high systolic BP, low diastolic BP, high fasting glucose, low hemoglobin, high TG, low total cholesterol, low HDL-cholesterol, low LDL-cholesterol, and high uric acid were associated with a low MMSE. Due to conducting *t*-tests and one-way analysis of variance (= general linear model) many times, the multiple-testing-adjusted corrections of *p* values were computed by stepdown Bonferroni (threshold of *p* = 0.0025; *p*0/N, *p*0 = 0.05, N = 20 independent variables).

The associations among MetS and its components with MMSE in multivariable linear regression analysis are shown in Table 4. After adjusting for age, sex, smoking history, regular exercise habits, education status, hemoglobin, total cholesterol, and uric acid (significant variables of Table 3 (*p* < 0.0025) except for diabetes, hypertension, diastolic BP, fasting glucose, TG, and HDL-cholesterol), MetS (*p* = 0.002), abdominal obesity (*p* < 0.001), low HDL-cholesterol (*p* = 0.004), and hyperglycemia (*p* = 0.012) were significantly associated with a low MMSE. However, hypertriglyceridemia (*p* = 0.097) and high BP (*p* = 0.684) were not associated with MMSE.

### 3.4. Association of Obesity-Related Indices and MMSE

The associations among the obesity-related indices with MMSE in multivariable linear regression analysis are shown in Table 5. Table 5 shows the association of obesity-related indices and MMSE using multivariable linear regression analysis in study participants. Different multivariable linear regression analyses were performed for different indices as follows:

Adjusted for age, sex, diabetes, hypertension, smoking history, regular exercise habits, education status, diastolic BP, fasting glucose, hemoglobin, TG, total cholesterol, HDL-cholesterol, and uric acid (significant variables of Table 3 (*p* < 0.0025)) for BMI, WHR, WHtR, BRI, CI, BAI, AVI, and ABSI.

Adjusted for age, sex, diabetes, hypertension, smoking history, regular exercise habits, education status, diastolic BP, fasting glucose, hemoglobin, triglyceride, total cholesterol, HDL-cholesterol, and uric acid (significant variables of Table 3 (*p* < 0.0025) except for TG) for LAP.

Adjusted for age, sex, diabetes, hypertension, smoking history, regular exercise habits, education status, diastolic BP, fasting glucose, hemoglobin, triglyceride, total cholesterol, HDL-cholesterol, and uric acid (significant variables of Table 3 (*p* < 0.0025) except for TG and fasting glucose) for TyG index.

After multivariable analysis, participants with high BMI (*p* = 0.001), high WHR (*p* < 0.001), high WHtR (*p* < 0.001), high BRI (*p* < 0.001), high CI (*p* < 0.001), high BAI (*p* < 0.001), high AVI (*p* < 0.001), and high ABSI (*p* < 0.001) were significantly associated with a low MMSE. However, LAP (*p* = 0.281) and TyG index (*p* = 0.082) were not associated with MMSE.

## 4. Discussion

The results of this large study show that MetS and its components (except hypertriglyceridemia and high BP) were associated with a low MMSE score. In addition, high values of obesity-related indices (BMI, AVI, BAI, WHR, ABSI, WHtR, CI, and BRI) were associated with a low MMSE score.

The first important finding of this study is that MetS was associated with poor cognitive function. Moreover, the number of MetS components increased with the severity of cognitive impairment. Several studies have suggested that MetS and its components may be associated with impaired cognitive function [32,33,34,35]. For example, a cross-sectional study conducted in China reported that MetS was associated with cognitive impairment as assessed using the MMSE, and that preventing MetS, and in particular hypertension and abdominal obesity, may help to prevent cognitive impairment [34]. In addition, in the French Three-City Study, the presence of MetS and several of its components (hypertriglyceridemia and low HDL-cholesterol) in older adults was inversely associated with global cognitive decline as assessed by a lower MMSE score. In addition, some subjects with hypertension or DM had a higher risk of a lower Benton Visual Retention Test score, which assesses visual working memory, and some subjects with low HDL-cholesterol and DM had a lower Isaacs Set Test score, which assesses verbal fluency [35]. The possible mechanisms underlying the association between MetS and poor cognitive function could be due to MetS-related carotid atherosclerosis with impaired cerebrovascular reactivity [36], increased carotid stiffness [37], and intima-media thickness [38] reducing blood supply to the central nervous system. Impaired vascular reactivity has been associated with insulin resistance and obesity-associated inflammation of the micro-vasculature, leading to lower efficiency of clearing metabolic “waste” products (such as carbon dioxide, excess lactate, other metabolites, and heat), and other potentially damaging effects such as hypothalamic-pituitary-adrenal axis dysregulation, and increased oxidative stress, which may impair brain function [39].

The second important finding of this study is that abdominal obesity and obesity-related indices were associated with poor cognitive function. Further, the prevalence of abdominal obesity and the values of obesity-related indices increased with the severity of cognitive impairment. A possible explanation for these findings is that obesity induces chronic, low-grade inflammation, which has been associated with insulin resistance [40]. Some studies have reported similar results to ours with regard to abdominal obesity [34,40,41]. A previous community-based cross-sectional study showed that the presence of abdominal obesity could predict a higher risk of cognitive impairment as assessed with a lower MMSE score [34]. Another study demonstrated inverse relationships between anthropometric measures of abdominal obesity, BMI, WC, and WHR with impaired cognitive function in middle-aged adults due to reduced neural integrity [40]. In addition, a large prospective longitudinal study of community-dwelling middle-aged to older adults reported similar results in that abdominal obesity, elevated TGs, and low HDL-cholesterol were associated with impaired cognitive performance [41]. Taken together, these findings indicate that easily calculated obesity-related indices could be used to survey and detect people at risk of cognitive impairment.

The third important finding of this study is that low HDL-cholesterol was associated with poor cognitive function. The Framingham Offspring Study found that the presence of abdominal obesity, elevated TGs, and low HDL-cholesterol was associated with a lower level of cognitive performance, which is partially consistent with our results [41]. Another cross-sectional study conducted in China found that TGs were significantly negatively associated with cognitive impairment, and that there was a significant linear trend between TG level and MMSE score [42]. A possible explanation is that these vascular risk factors may increase the risk of brain injury (e.g., stroke), thereby causing cognitive impairment [41]. In addition, HDL-cholesterol functions by bringing other types of cholesterol from tissues (including the brain) to the liver for metabolism, and it also enters the blood–brain barrier to regulate the metabolism of amyloid β, a major constituent of amyloid plaque. Therefore, a lower HDL-cholesterol level may induce the deposition of abnormal metabolites, leading to impaired cognitive function [43]. Furthermore, higher TG might increase the circulating complex of amyloid β synthesized in enterocytes and triglyceride-rich lipoproteins, which would impact the blood–brain barrier and subsequently increase brain amyloid deposition and result in cerebral amyloidosis [44]. However, a cross-sectional study in Japan showed no relationship between hypertriglyceridemia and cognitive impairment [19] and a study in China concluded that high normal TG had a significant and beneficial effect on cognitive function [42]. The possible explanation was that recent molecular biology studies showing that TGs can increase the blood–brain barrier transport of ghrelin and insulin and raising serum TG levels could also affect the expression of orexigenic hypothalamic peptides, which might improve the cognitive function [42]. In addition, TG may be a meaningful indicator of nutrition status, so high normal levels of TG may demonstrate an adequate but not excessive intake of nutrients and energy, which would be helpful in maintaining brain function [42]. In our study, hypertriglyceridemia did not reach significant levels. This was possibly because our participants had good control of TG levels causing the insignificant difference.

We also found an association between hyperglycemia and a low MMSE score, and the prevalence of hyperglycemia increased with the severity of cognitive impairment. In addition, the severity of cognitive impairment increased with the prevalence of hyperglycemia, which is consistent with previous studies [34,45]. A previous study conducted in rural China reported an association between hyperglycemia and an increased risk of cognitive impairment, where cognitive impairment was defined as an MMSE < 27 [34]. Another study also concluded that hyperglycemia significantly exacerbated cognitive impairment in frail and hypertensive adults [45]. A possible explanation for these findings is that hyperglycemia is associated with cellular insulin resistance [46], which can further increase the risk of cardiovascular disease [47]. Insulin receptor signaling in the central nervous system has been shown to play important roles in the regulation of neurodegeneration, synaptogenesis, learning, and memory, and insulin has also been shown to play an important role in promoting the recovery of neurons after injury through the activation of survival pathways [48]. In addition, hyperglycemia has been shown to impair endothelial function independently of the presence of DM, and to be a predictor of severe cardiovascular outcomes [45]. Moreover, type 2 DM may also affect cognition as it is considered to be a risk factor for cardiovascular disease [49]. Type 2 DM has also been associated with global brain atrophy and higher vascular brain lesion load compared to people without DM [50,51]. In a review, the pathogenesis between hyperglycemia and cognitive impairment were also well discussed. It was indicated that hyperglycemia was involved with disrupted glucose transport leading to the dysregulation of osmolarity and brain metabolism, greater atrophy of the hippocampus and amygdala with elevating glucose level, dysfunctional glucose regulation eliciting neuronal synaptic reorganization, decreased insulin secretion or action, impairment in the hypothalamic−pituitary−adrenal (HPA) axis, obesity, hyperleptinemia, oxidative stress, and inflammation. In detail, impairment in the HPA axis with chronic elevation of corticosteroid levels inducing changes in transcription of genes encoding corticotropin-releasing hormone receptors suppresses dendritic arborization, and inhibits neurogenesis in the hippocampus. Furthermore, cortical, subcortical, and hippocampal atrophy (particularly in the dentate gyrus) has also been detected in type 2 diabetic patients by brain magnetic resonance imaging, which was also associated with cognitive impairments [52].

We did not observe a significant association between high BP and MMSE score. This is in contrast to a previous cross-sectional study, which reported that an elevated BP was associated with a higher risk of cognitive impairment [34]. The authors suggested that the mechanism may be through a high BP facilitating the production of reactive oxygen species, leading to cerebral vessel inflammation and cerebral blood flow dysregulation [34]. However, the Longitudinal Aging Study Amsterdam enrolled 1183 participants aged 65–88 years, and revealed no relationship between hypertension and cognitive function as assessed using the MMSE [53]. In addition, a cross-sectional study also found no significant difference in the prevalence of cognitive impairment in older adults with and without hypertension [54]. Moreover, a cross-sectional study from the Kungshohnen project reported an inverse relationship between systolic and diastolic BPs with the prevalence of dementia in older adults [55]. In addition, a population-based cohort study suggested that orthostatic hypotension, a condition reflecting insufficient hemodynamic adaptation to postural changes, which is common in older adults, was associated with an increased risk of dementia and faster progression from no dementia to dementia [56]. Moreover, the Bronx Aging Study, which enrolled community-dwelling volunteers with 21 years of follow-up data, demonstrated that a low BP was significantly associated with a higher risk of dementia [57]. In particular, those with a persistently low BP for over 2 years had an appropriately two-fold increased risk of developing dementia [57]. The authors hypothesized that the link between hypotension and cognitive impairment may be through cerebral hypoperfusion caused by low BP leading to the overexpression of β-amyloid precursor protein, thereby causing vascular endothelial damage and an increased risk of dementia [57]. The insignificant association between BP and cognitive function in our study may be because our participants had good BP control.

In recent years, there have been several studies about the relationship between MetS and depression. A systematic review and meta-analysis study, recruiting seventeen observational studies, indicated that there was a significant relationship between depression and MetS [58]. In addition, a three-city study pointed out that MetS was associated with an almost doubling of the odds for new-onset depressive symptoms, which was evaluated with the Center for Epidemiologic Studies Depression Scale in the age group 65–70 years, and also indicated that MetS and depression comprised a bidirectional relationship [59]. Furthermore, the authors of a study of the secondary analysis of a randomized controlled trial, involving participants aged 60 and older with major depressive disorder, came to the the conclusion that MetS was associated with greater symptom severity and longer duration of depression by assessing with Montgomery–Asberg Depression Rating Scale [60]. In addition, MetS patients need longer antidepressant medication to relieve depression symptoms [60]. The possible mechanisms for MetS patients being prone to having depression were that physical limitations or social stigma caused by obesity may predispose them to depression [61]. In addition, raising the level of inflammatory cytokines such as interleukein 6, C-reactive protein, and leptin resistance may also be involved with depression [62]. Furthermore, patients with MetS often have cardiovascular problems that can also lead to vascular damage in the brain and lead to depression [62]. The relationship between depression and cognitive dysfunction was also studied. A review indicated that it was well established that patients with major depressive disorder may experience pronounced cognitive dysfunction among elderly adults and depression affecting cognition in multiple domains even among younger age groups [63]. In addition, a systematic review and meta-analysis also indicated that depression was associated with significant deficits across all tasks within the domains of executive function, memory, and attention, by evaluating with a single neuropsychological test battery [64]. However, there was no significant correlation between MetS and depression in our study, possibly because the depression in this article was self-reported rather than being diagnosed by a physician and there was no history of medication for depression, thus affecting the results.

The main strengths of our study include the large community-based cohort of healthy participants with no history of cancer, comprehensive neurocognitive assessments using the MMSE, and controlling for confounding factors including lifestyle habits and cardiovascular risk factors. However, there are also several limitations. First, the use of medications that may affect MetS, fasting glucose, BP, and lipid profile such as hypnotics, lipid-lowering agents, antihypertensive medications, and anti-diabetic agents is not recorded in the TWB. This may have led to an underestimation of the risk of MCI and the association between MetS and cognitive function. Second, due to the cross-sectional nature of this study, we could not explore causal relationships. Further longitudinal studies are warranted to investigate the risk of incident dementia. Third, we did not adjust the MMSE score for sensory impairment. Fourth, all participants in this study were of Chinese ethnicity, which may limit the generalizability of our results. Finally, cognitive performance was assessed using only the MMSE, which may have affected the results. Although the MMSE has some limitations, its use as a brief screening test to quantitatively evaluate the severity of cognitive impairment and document changes over time has been validated [65].

In conclusion, we found that MetS and its components (except hypertriglyceridemia and high BP) were associated with poor cognitive function. In addition, high obesity-related index values, including BMI, AVI, BAI, WHR, ABSI, WHtR, CI, and BRI, were associated with poor cognitive function. Moreover, the prevalence of MetS and the values of the obesity-related indices increased with the severity of cognitive impairment.

## Figures and Tables

**Table 1:** Comparison of clinical characteristics among participants according to total MMSE scores ≥ 24 or <24.

Characteristics	MMSE ≥ 24 (*n* = 26,358)	MMSE < 24 (*n* = 2128)	*p*
MMSE (score)	27.9 ± 1.7	21.0 ± 2.5	<0.001
Age (year)	63.9 ± 2.9	64.5 ± 2.9	<0.001
Male sex (%)	40.1	28.6	<0.001
DM (%)	10.6	14.1	<0.001
Hypertension (%)	24.8	30.4	<0.001
Smoking history (%)	26.0	19.7	<0.001
Alcohol history (%)	8.9	8.2	0.242
Regular exercise habits (%)	63.7	57.3	<0.001
Depression history (%)	3.8	4.1	0.572
Education status			<0.001
Lower than elementary school (%)	12.2	61.8	
Middle and high school (%)	43.1	30.6	
Higher than college (%)	44.8	7.6	
Living alone (%)	9.4	8.4	0.106
SBP (mmHg)	130.5 ± 19.3	131.3 ± 19.6	0.053
DBP (mmHg)	75.6 ± 10.9	74.6 ± 10.9	<0.001
Body height (cm)	159.6 ± 7.8	156.6 ± 7.1	<0.001
Body weight (kg)	62.0 ± 10.8	61.7 ± 10.0	0.141
Waist circumference (cm)	85.0 ± 9.4	86.8 ± 9.6	<0.001
Hip circumference (cm)	95.0 ± 6.4	95.5 ± 6.7	<0.001
Laboratory parameters			
Fasting glucose (mg/dL)	100.8 ± 22.3	103.9 ± 30.3	<0.001
Hemoglobin (g/dL)	13.9 ± 1.3	13.6 ± 1.4	<0.001
Triglyceride (mg/dL)	119.8 ± 81.7	123.9 ± 74.9	<0.001
Total cholesterol (mg/dL)	199.9 ± 36.5	199.2 ± 36.8	0.424
HDL-cholesterol (mg/dL)	54.3 ± 13.5	53.7 ± 13.2	0.026
LDL-cholesterol (mg/dL)	123.2 ± 31.9	122.4 ± 32.2	0.277
eGFR (mL/min/1.73 m^2^)	93.9 ± 21.9	94.3 ± 24.6	0.385
Uric acid (mg/dL)	5.6 ± 1.4	5.6 ± 1.4	0.174
MetS (%)	32.1	39.9	<0.001
MetS component			
Abdominal obesity (%)	52.9	65.5	<0.001
Hypertriglyceridemia (%)	22.8	24.1	0.167
Low HDL-cholesterol (%)	25.6	31.2	<0.001
Hyperglycemia (%)	34.1	37.9	<0.001
High blood pressure (%)	57.6	61.0	0.002
Obesity-related indices			
BMI (kg/m^2^)	24.3 ± 3.4	25.1 ± 3.4	<0.001
WHR (%)	89.4 ± 6.5	90.8 ± 6.8	<0.001
WHtR (%)	53.3 ± 5.8	55.5 ± 6.3	<0.001
BRI	4.1 ± 1.2	4.5 ± 1.4	<0.001
CI	1.25 ± 0.08	1.27 ± 0.09	<0.001
BAI	29.3 ± 4.1	3.9 ± 4.4	<0.001
AVI	14.7 ± 3.2	15.3 ± 3.3	<0.001
ABSI	0.080 ± 0.005	0.081 ± 0.005	<0.001
LAP	34.5 ± 30.5	39.1 ± 30.9	<0.001
TyG index	8.5 ± 0.6	8.6 ± 0.6	<0.001

Abbreviations: MMSE, Mini-Mental State Examination; DM, diabetes mellitus; SBP, systolic blood pressure; DBP, diastolic blood pressure; MetS, metabolic syndrome; HDL, high-density lipoprotein; LDL, low-density lipoprotein; eGFR, estimated glomerular filtration rate; FVC, forced vital capacity, FEV1, forced expiratory volume in 1 s; BMI, body mass index; WHR, waist–hip ratio; WHtR, waist-to-height ratio; BRI, body roundness index; CI, conicity index; BAI, body adiposity index; AVI, abdominal volume index; VAI, visceral adiposity index; ABSI, a body shape index; LAP, lipid accumulation product; TyG index, triglyceride glucose index.

**Table 2:** The prevalence of MetS and its components, and the values of obesity-related indices according to the severity of cognitive function in study participants.

Variables	MMSE ≥ 24 (*n* = 26,358)	MMSE 18–23 (*n* = 1939)	MMSE 0–17 (*n* = 189)	*p*
MetS (%)	32.1	39.8 *	41.3 *	<0.001
MetS numbers	1.9 ± 1.3	2.2 ± 1.3 *	2.3 ± 1.3 *	<0.001
MetS component				
Abdominal obesity (%)	52.9	65.2 *	68.3 *	<0.001
Hypertriglyceridemia (%)	22.8	24.3	22.2	0.312
Low HDL-cholesterol (%)	25.6	30.8 *	34.9 *	<0.001
Hyperglycemia (%)	34.1	37.5 *	41.3	0.001
High blood pressure (%)	57.6	61.2 *	58.7	0.008
Obesity-related indices				
BMI (kg/m^2^)	24.3 ± 3.4	25.1 ± 3.4 *	25.3 ± 3.6 *	<0.001
WHR (%)	89.4 ± 6.5	90.7 ± 6.7 *	91.7 ± 7.7 *	<0.001
WHtR (%)	53.3 ± 5.8	55.4 ± 6.2 *	56.9 ± 6.9 *^†^	<0.001
BRI	4.1 ± 1.2	4.5 ± 1.3 *	4.8 ± 1.5 *^†^	<0.001
CI	1.25 ± 0.08	1.27 ± 0.08 *	1.29 ± 0.10 *^†^	<0.001
BAI	29.3 ± 4.1	30.8 ± 4.4 *	31.9 ± 4.6 *^†^	<0.001
AVI	14.7 ± 3.2	15.3 ± 3.3 *	15.7 ± 3.7 *	<0.001
ABSI	0.080 ± 0.005	0.081 ± 0.005 *	0.082 ± 0.006 *^†^	<0.001
LAP	34.5 ± 30.5	39.0 ± 30.9 *	40.3 ± 31.4 *	<0.001
TyG index	8.5 ± 0.6	8.6 ± 0.6 *	8.6 ± 0.5	<0.001

Abbreviations are the same as in Table 1. * *p* < 0.05 compared with MMSE ≥ 24; ^†^ *p* < 0.05 compared with MMSE 18–23.

**Table 3:** Determinants for MMSE using univariable linear regression analysis in study participants.

Characteristics	Univariable	
	Unstandardized Coefficient β (95% Confidence Interval)	*p*
Age (per 1 year)	−0.077 (−0.087, −0.067)	<0.001
Male (vs. female)	0.299 (0.240, 0.359)	<0.001
DM	−0.404 (−0.498, −0.311)	<0.001
Hypertension	−0.240 (−0.307, −0.173)	<0.001
Smoking history	0.157 (0.090, 0.224)	<0.001
Alcohol history	0.031 (−0.072, 0.134)	0.553
Regular exercise habits	0.235 (0.174, 0.295)	<0.001
Depression history	−0.052 (−0.204, 0.100)	0.502
Education status		
Lower than elementary school	Reference	
Middle and high school	2.520 (2.444, 2.596)	<0.001
Higher than college	3.402 (3.325, 3.478)	<0.001
Living alone	0.112 (0.011, 0.212)	0.029
SBP (per 1 mmHg)	−0.002 (−0.004, −0.001)	0.006
DBP (per 1 mmHg)	0.006 (0.003, 0.009)	<0.001
Laboratory parameters		
Fasting glucose (per 1 mg/dL)	−0.006 (−0.007, −0.005)	<0.001
Hemoglobin (per 1 g/dL)	0.103 (0.082, 0.125)	<0.001
Triglyceride (per 10 mg/dL)	−0.007 (−0.010, −0.003)	<0.001
Total cholesterol (per 10 mg/dL)	0.014 (0.006, 0.022)	<0.001
HDL-cholesterol (per 1 mg/dL)	0.006 (0.004, 0.008)	<0.001
LDL-cholesterol (per 1 mg/dL)	0.001 (0, 0.002)	0.004
eGFR (per 1 mL/min/1.73 m^2^)	0.001 (−0.001, 0.002)	0.294
Uric acid (per 1 mg/dL)	−0.042 (−0.064, −0.021)	<0.001

Values expressed as unstandardized coefficient β and 95% confidence interval. Abbreviations are the same as in Table 1.

**Table 4:** Association of MetS and its components with MMSE using multivariable linear regression analysis in study participants.

MetS and Its Components	Multivariable	
	Unstandardized Coefficient β (95% Confidence Interval)	*p*
MetS	−0.089 (−0.146, −0.031)	0.002
MetS component		
Abdominal obesity (%)	−0.136 (−0.111, 00.081)	<0.001
Hypertriglyceridemia (%)	−0.054 (−0.118, 0.010)	0.097
Low HDL-cholesterol (%)	−0.091 (−0.152, −0.029)	0.004
Hyperglycemia (%)	−0.071 (−0.127, −0.015)	0.012
High blood pressure (%)	0.011 (−0.043, 0.065)	0.684

Values expressed as unstandardized coefficient β and 95% confidence interval. Abbreviations are the same as in Table 1. Adjusted for age, sex, smoking history, regular exercise habits, education status, hemoglobin, total cholesterol, and uric acid (significant variables of Table 3 (*p* < 0.0025) except for diabetes, hypertension, diastolic blood pressure, fasting glucose, triglyceride, and HDL-cholesterol).

**Table 5:** Association of obesity-related indices with MMSE using multivariable linear regression analysis in study participants.

Obesity-Related Indices	Multivariable	
	Unstandardized Coefficient β (95% Confidence Interval)	*p*
BMI (per 1 kg/m^2^) ^a^	−0.015 (−0.023, −0.006)	0.001
WHR (per 1%) ^a^	−0.015 (−0.020, −0.011)	<0.001
WHtR (per 1%) ^a^	−0.016 (−0.021, −0.011)	<0.001
BRI (per 1) ^a^	−0.076 (−0.100, −0.052)	<0.001
CI (per 0.1) ^a^	−0.086 (−0.119. −0.052)	<0.001
BAI (per 1) ^a^	−0.015 (−0.022, −0.007)	<0.001
AVI (per 1) ^a^	−0.019 (−0.029, −0.010)	<0.001
ABSI (per 0.01) ^a^	−0.110 (−0.164, −0.056)	<0.001
LAP (per 1) ^b^	−0.001 (−0.002, 0)	0.281
TyG index (per 1) ^c^	−0.055 (−0.116, 0.007)	0.082

Values expressed as unstandardized coefficient β and 95% confidence interval. Abbreviations are the same as in Table 1. ^a^ Adjusted for age, sex, diabetes, hypertension, smoking history, regular exercise habits, education status, diastolic blood pressure, fasting glucose, hemoglobin, triglyceride, total cholesterol, HDL-cholesterol, and uric acid (significant variables of Table 3). ^b^ Adjusted for age, sex, diabetes, hypertension, smoking history, regular exercise habits, education status, diastolic blood pressure, fasting glucose, hemoglobin, triglyceride, total cholesterol, HDL-cholesterol, and uric acid (significant variables of Table 3 except for triglyceride). ^c^ Adjusted for age, sex, diabetes, hypertension, smoking history, regular exercise habits, education status, diastolic blood pressure, fasting glucose, hemoglobin, triglyceride, total cholesterol, HDL-cholesterol, and uric acid (significant variables of Table 3 except for triglyceride and fasting glucose).

## Data Availability

The data underlying this study are from the Taiwan Biobank. Due to restrictions placed on the data by the Personal Information Protection Act of Taiwan, the minimal data set cannot be made publicly available. Data may be available upon request to interested researchers. Please send data requests to S.-C.C., Division of Nephrology, Department of Internal Medicine, Kaohsiung Medical University Hospital, Kaohsiung Medical University.
